# Development and initial implementation of electronic clinical decision supports for recognition and management of hospital-acquired acute kidney injury

**DOI:** 10.1186/s12911-020-01303-x

**Published:** 2020-11-04

**Authors:** Megan Howarth, Meha Bhatt, Eleanor Benterud, Anna Wolska, Evan Minty, Kyoo-Yoon Choi, Andrea Devrome, Tyrone G. Harrison, Barry Baylis, Elijah Dixon, Indraneel Datta, Neesh Pannu, Matthew T. James

**Affiliations:** 1 Department of Medicine, Cumming School of Medicine, University of Calgary, 3280 Hospital Drive NW, Calgary, AB T2N 4Z6 Canada; 2 Department of Community Health Sciences, Cumming School of Medicine, University of Calgary, Calgary, AB Canada; 3 Alberta Health Services, Calgary, AB Canada; 4 Department of Surgery, Cumming School of Medicine, University of Calgary, Calgary, AB Canada; 5 Department of Medicine, Faculty of Medicine and Dentistry, University of Alberta, Edmonton, AB Canada; 6 O’Brien Institute for Public Health, Cumming School of Medicine, University of Calgary, Calgary, AB Canada; 7 Libin Cardiovascular Institute, Cumming School of Medicine, University of Calgary, Calgary, AB Canada

**Keywords:** Clinical decision support, Electronic medical record, Acute kidney injury

## Abstract

**Background:**

Acute kidney injury (AKI) is common in hospitalized patients and is associated with poor patient outcomes and high costs of care. The implementation of clinical decision support tools within electronic medical record (EMR) could improve AKI care and outcomes. While clinical decision support tools have the potential to enhance recognition and management of AKI, there is limited description in the literature of how these tools were developed and whether they meet end-user expectations.

**Methods:**

We developed and evaluated the content, acceptability, and usability of electronic clinical decision support tools for AKI care. Multi-component tools were developed within a hospital EMR (Sunrise Clinical Manager™, Allscripts Healthcare Solutions Inc.) currently deployed in Calgary, Alberta, and included: AKI stage alerts, AKI adverse medication warnings, AKI clinical summary dashboard, and an AKI order set. The clinical decision support was developed for use by multiple healthcare providers at the time and point of care on general medical and surgical units. Functional and usability testing for the alerts and clinical summary dashboard was conducted via in-person evaluation sessions, interviews, and surveys of care providers. Formal user acceptance testing with clinical end-users, including physicians and nursing staff, was conducted to evaluate the AKI order set.

**Results:**

Considerations for appropriate deployment of both non-disruptive and interruptive functions was important to gain acceptability by clinicians. Functional testing and usability surveys for the alerts and clinical summary dashboard indicated that the tools were operating as desired and 74% (17/23) of surveyed healthcare providers reported that these tools were easy to use and could be learned quickly. Over three-quarters of providers (18/23) reported that they would utilize the tools in their practice. Three-quarters of the participants (13/17) in user acceptance testing agreed that recommendations within the order set were useful. Overall, 88% (15/17) believed that the order set would improve the care and management of AKI patients.

**Conclusions:**

Development and testing of EMR-based decision support tools for AKI with clinicians led to high acceptance by clinical end-users. Subsequent implementation within clinical environments will require end-user education and engagement in system-level initiatives to use the tools to improve care.

## Background

Acute kidney injury (AKI) is a common complication in hospitalized patients that is associated with poor patient outcomes and high costs of care [1, 2]. The recognition and initial management of AKI in hospital usually depends on the awareness and judgment of nursing staff and physicians. However, it has been well described that AKI often initially goes unrecognized and that many care providers lack knowledge and confidence about appropriate management for AKI [1, 3–5]. The implementation of electronic clinical decision support (CDS) tools within electronic medical record (EMR) systems has been widely suggested to improve AKI recognition and care [1, 2, 6, 7].

Several studies have reported on implementation of AKI alerts in acute care settings. A recent systematic review identified 16 studies that implemented electronic alerts for AKI, including both interruptive and non-disruptive alert systems [8]. Interruptive alerts are typically pushed to healthcare providers through various modes (e.g. text message, pager alert, EMR pop-up) whereas non-disruptive alerts require provider actions (e.g. navigate to click and view alert on EMR). The impact of alerts on clinical responses to AKI was variable across studies, although few of these studies linked alerts with accompanying guidance on management after the alert was received [9–13]. While CDS tools appear to have potential to enhance recognition of AKI at its early stages, their impact on clinical care and outcomes among hospitalized patients has been inconsistent. The design features of CDS systems in other areas of clinical practice have been shown to be critical to their success [14, 15]. With the variable evidence in support of CDS tools for AKI care, considerate design of CDS tools for AKI could help optimize their potential to improve care and outcomes once implemented.

With this in mind, we applied knowledge on best practices for CDS tools to develop, test, refine, and deploy a novel suite of AKI CDS tools in an existing hospital EMR system. Given that clinical decision support tools are most effective when they provide actionable information beyond assessments [14], we specifically sought to link the design of electronic alerts for AKI recognition to associated decision support tools that would guide appropriate management. Here, we describe the iterative process of development and testing of these electronic CDS tools for AKI.

## Methods

**Fig. 1:**
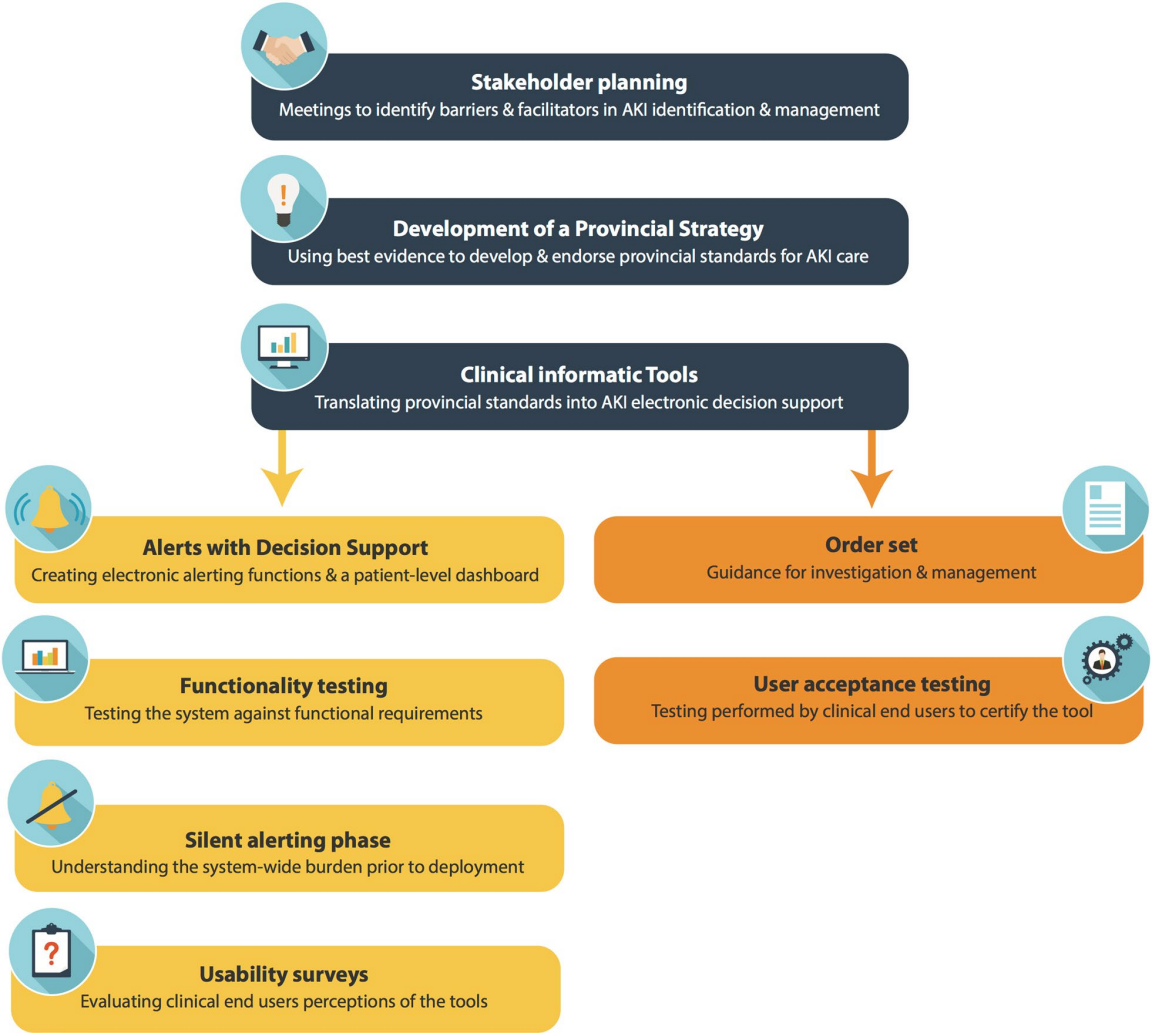
Development process for acute kidney injury clinical decision support system within the province of Alberta, Canada. AKI: Acute Kidney Injury. Images used under permission: edel/Shutterstock.com

A multi-phase process was used to develop and test an electronic clinical decision support system for AKI care in Calgary, Alberta, Canada. The first phase involved engagement with multidisciplinary stakeholders to develop a provincial strategy for AKI care based on clinical practice guidelines and identify content for clinical decision support. The second phase included development of an AKI alert function prototype, a clinical summary dashboard, and an order set within the hospital EMR system. The last phase of development included testing and refinement of the tools using feedback from clinical end-users. Figure 1 displays the development process for this intervention. The study was approved by the University of Calgary Conjoint Health Research Ethics Board (REB14-1531).

### Stakeholder engagement and identification of content for AKI clinical decision support tools

A multidisciplinary team representing CDS developers and end-users, including physicians, nurses, pharmacists, health services researchers, and clinical informatics specialists assembled in a working group to guide the content and features of the CDS. Content and development were guided by a previous planning meeting that identified knowledge gaps and areas for improvement in AKI care [1], and from a modified Delphi process to establish local stakeholder consensus on quality indicators for AKI care [16]. Content was informed from a review of international guidelines for AKI [6, 17, 18], as well as identified publications on CDS for AKI [9, 19–34]. A clinical knowledge topic and pathway for AKI recognition, clinical assessment, and management was developed by the workgroup, followed by a review by representatives from Alberta Health Services Clinical Support Services. Recommendations from stakeholder review were incorporated into the final content document—an AKI Clinical Knowledge Topic accessible to Alberta Health Services clinical staff.

The content was translated into requirements for AKI CDS in the hospital EMR system, Sunrise Clinical Manager™ (SCM™, Allscripts Healthcare Solutions, Inc.), currently in use in acute care settings in Calgary, Alberta. The Calgary Zone Alberta Health Services Clinical Decision Support Configuration and Development Team reviewed the content and objectives and developed prototypes of the CDS tools in SCM™. Development involved an iterative process that included the development of prototypes, review of their design by the AKI workgroup, functionality testing, and refinement before the final approval and deployment in the SCM™ EMR system.

### Development of the electronic AKI clinical decision support tools

All of the electronic CDS tools were developed within the SCM™ hospital EMR system, which was in use across all hospitals in Calgary, Alberta at the time of development. The existing EMR system was selected to integrate the electronic tools within usual care processes. Tools were programed into SCM™ using the Medical Logic Module (MLM) editor. The CDS included a collection of four electronic tools designed to support AKI recognition and early management. The first were AKI Stage Alerts to signal healthcare providers of AKI onset and its severity, along with a list of the patient’s active medications that may contribute to AKI. The second alert was an Adverse AKI Medication Warning to notify ordering physicians when medications that could reduce kidney function are ordered for patients with existing AKI. The third tool was an AKI Clinical Summary Dashboard to provide an overview of relevant clinical measurements for patients with AKI, including changes in serum creatinine, information to support assessment of volume status and administration of intravenous fluids, and medications that contribute to AKI or are renally eliminated. Lastly, we developed an AKI Order Set to guide the ordering of diagnostic tests, initiating therapies including intravenous fluids and diuretics, medication management and safety, and guidance for consultation with specialists.

### AKI stage alerts

**Fig. 2:**
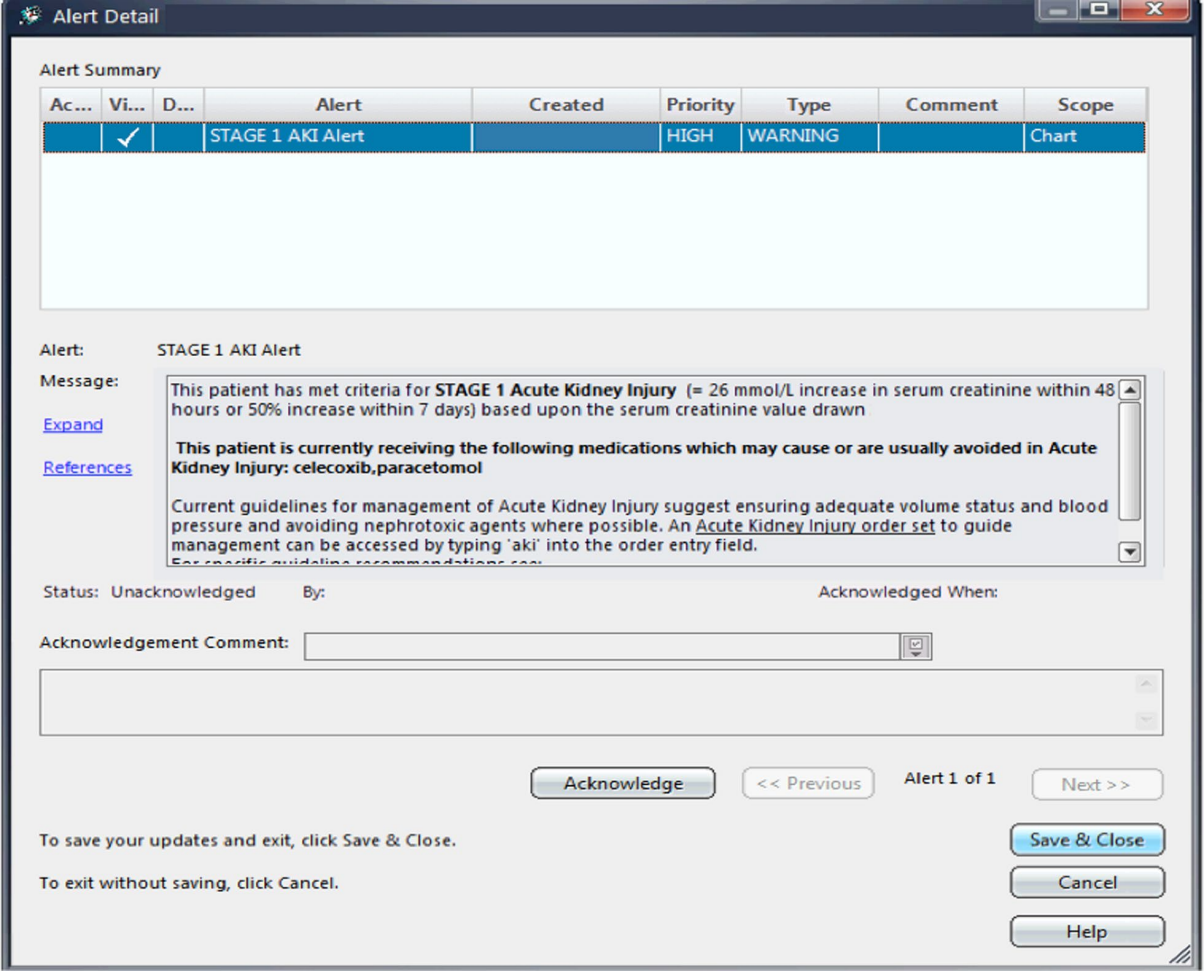
Acute kidney injury stage alert

The AKI stage alerts were generated based on changes in serum creatinine results, in accordance with the AKI stage criteria of the International Kidney Disease Improving Global Outcomes (KDIGO) clinical practice guideline for AKI [18]. AKI was ascertained in the system using the National Health Service England algorithm [35], such that the most recent hospital blood work is used for the index value and the system scans the patient’s records up to one year prior to obtain a baseline value. The AKI stage alert was generated once when the KDIGO criteria for any AKI stage are first met, and new alerts were generated for higher AKI stages if AKI progression occurred. If a patient recovered from AKI, defined by a subsequent creatinine below the threshold required for a Stage 1 AKI alert, but has another creatinine result that meets criteria for a second event, the AKI stage alert appeared again for the same patient. Therefore, a patient could have multiple AKI stage alerts during their hospital stay (Fig. 2).

Alert fatigue and accessibility were key considerations in the design of the display of alerts in the EMR. Alerts were non-interruptive and appeared as a red flag adjacent to the patient name on the main view of the EMR. This allowed providers to access alert information at natural task boundaries, instead of contributing to cognitive overload through disruption of the ‘origin task’ with an alert. When the flag was selected by the healthcare provider, the alert pop-up displayed the patient’s stage of AKI and a list of the patient’s active medications at the time of the alert that may cause AKI. The alert pop-up also included an ‘acknowledge’ button. The AKI alert was designed to appear on the front screen of the EMR for all users until any user acknowledged it, which removed it from display on the front screen. AKI alerts were permanently retained in other sections of the EMR, including the patient summary section under alerts, and on the AKI clinical summary dashboard. The alerts were integrated with the other CDS tools by adding information on accessing the AKI order set and providing a link to the AKI Clinical Knowledge Topic to aid healthcare providers’ access knowledge for management of AKI. Table 1 summarizes the design features of the AKI stage alert and rationale for these elements.

**Table 1:** Acute kidney injury alerts design and rationale

Design features	Rationale
Criteria for alert	Change in serum creatinine based on KDIGO criteria, employing the National Health Service England algorithm. The change in creatinine between the reference value (measured in hospital) and the baseline value taken from the prior 7 days if available, and if not available, then a median of all values from one year prior to the reference value
Non-interruptive	Alerts are non-interruptive to avoid alert fatigue from multiple disruptive notifications to healthcare providers
Available to all	Alerts are available to all healthcare providers due to their diverse roles on the units and to allow for a concerted response by the care team for managing AKI
Alerts deployed at specific locations	The surgical units where the alerts are deployed were chosen based on their high incidence of AKI (identified through preliminary work) and the main initial management responses for AKI related to therapy with fluids and management of medications

*AKI* Acute kidney injury, *KDIGO* Kidney Disease Improving Global Outcomes

### Adverse AKI medication warnings

**Fig. 3:**
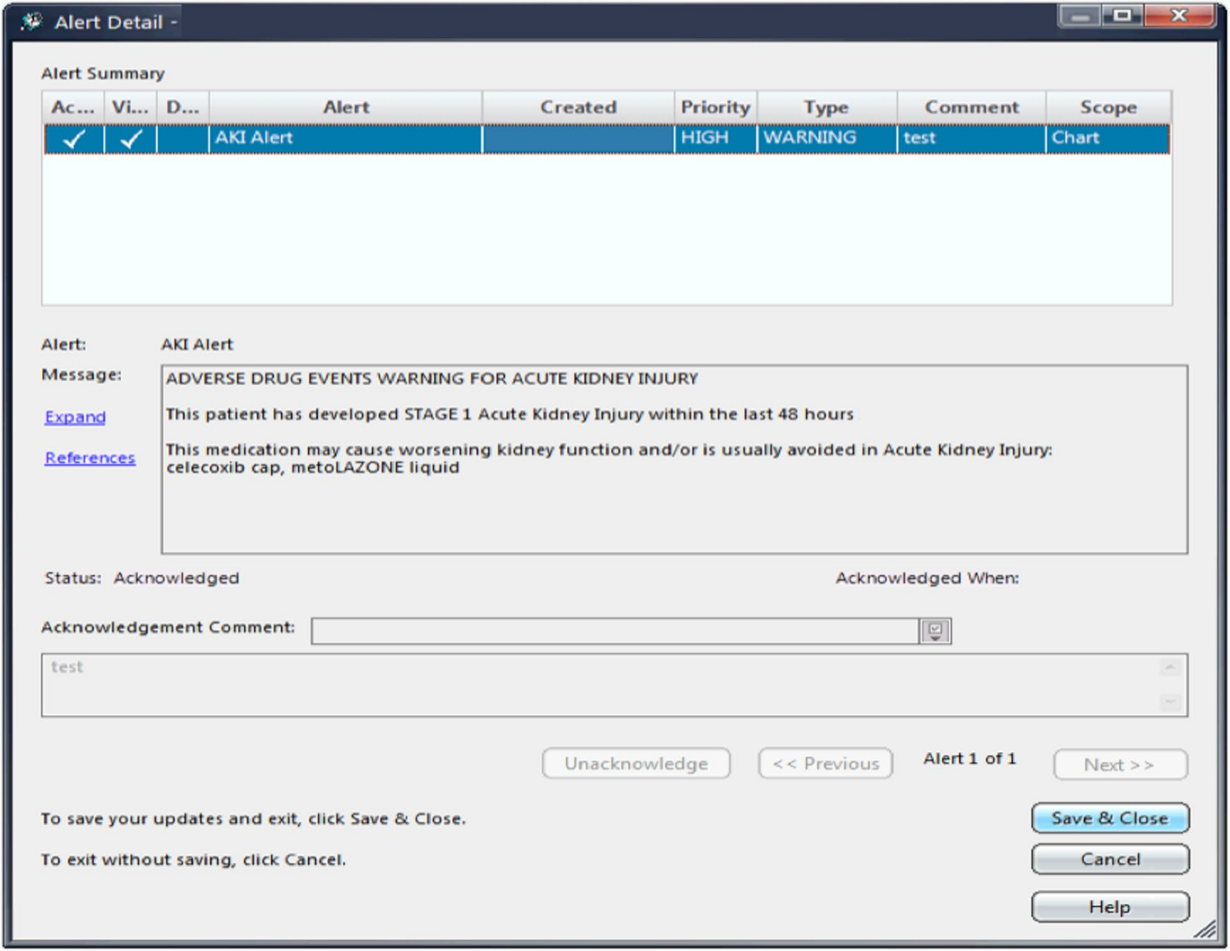
Acute kidney injury adverse medication warning

The adverse AKI medication warning was an alert generated when a medication that may reduce kidney function was ordered for a patient with AKI within 48 h of AKI onset (Fig. 3). An interruptive alert was generated within the ordering interface at the time of attempted order entry that identified the stage of AKI and listed the medications that triggered the warning. Medications that generate this alert were selected based on the work on medication safety in AKI previously published by McCoy et al. [28] and adapted to include the specific medications from the Alberta Health Services provincial hospital formularies. A list of included medications is provided in Additional file 1: Table S1.

An interruptive alert was deemed acceptable as the risk of alert fatigue was felt to be low in this scenario, since the number of alerts was anticipated to be few, and alerts would occur at the moment of order entry and not interrupt other tasks. The adverse AKI medication warnings were designed so that healthcare providers must acknowledge the alert, however they could still override the alert and proceed to complete the order entry of the medication if they desired.

### AKI clinical summary dashboard

**Fig. 4:**
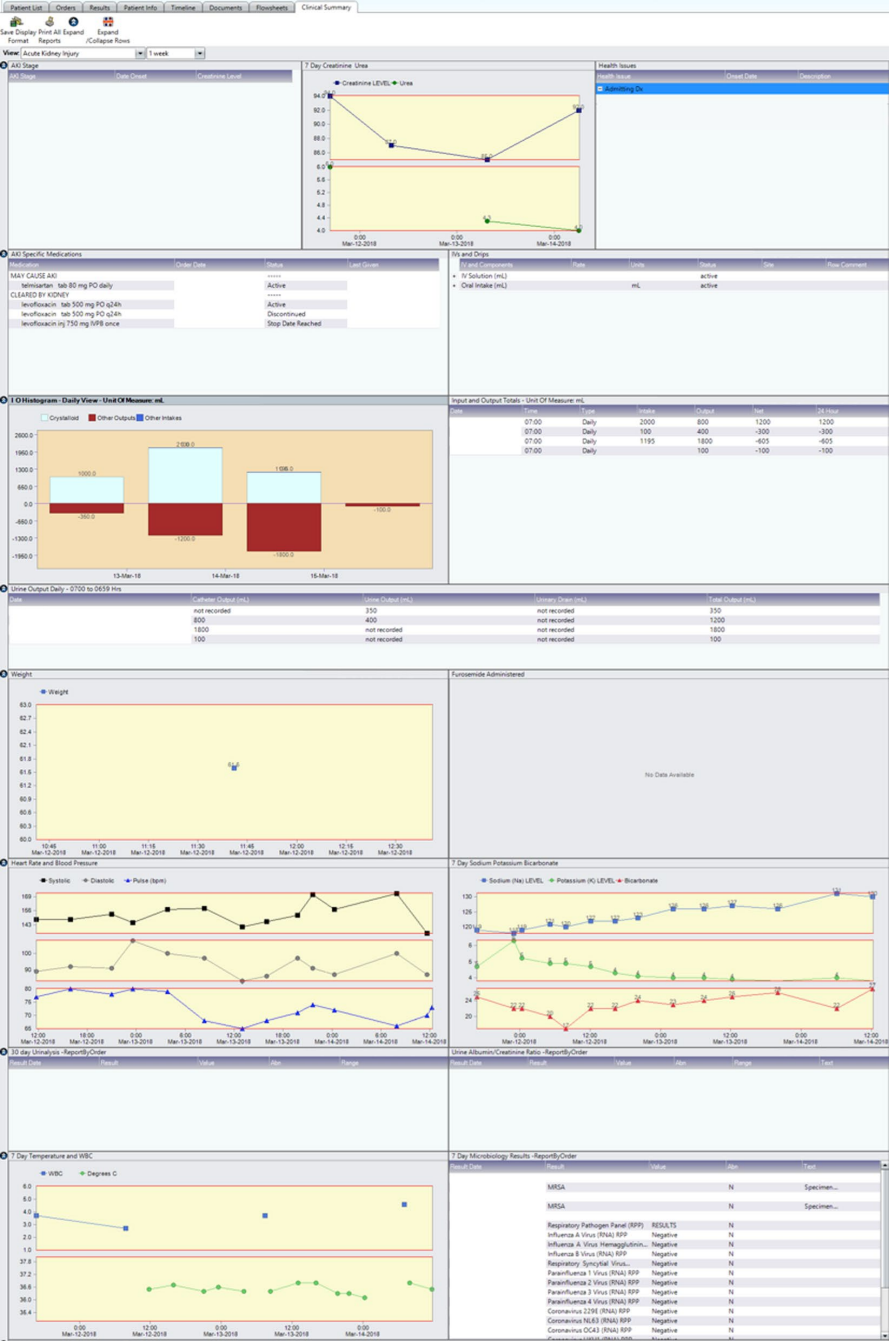
Acute kidney injury clinical summary dashboard

The clinical summary dashboard displayed patient information relevant to the clinical assessment of AKI, including the history of AKI stage alerts, display of temporal trends in serum creatinine, electrolyte, and urea levels, a listing of ordered medications that may worsen AKI and of renally cleared medications that carry a risk of accumulation and adverse medication safety events (Fig. 4). In order to support assessment of volume status and fluid therapies, the AKI dashboard also summarized urine output, fluid balance, and intravenous therapies. Furthermore, the dashboard view incorporated a display of vital signs (heart rate and blood pressure), other laboratory test results relevant to AKI urinalysis results, and urine protein tests) and sepsis (white blood cell count and microbiology cultures). Visit history and active health issues were also included (Fig. 4).

### AKI order set

**Fig. 5:**
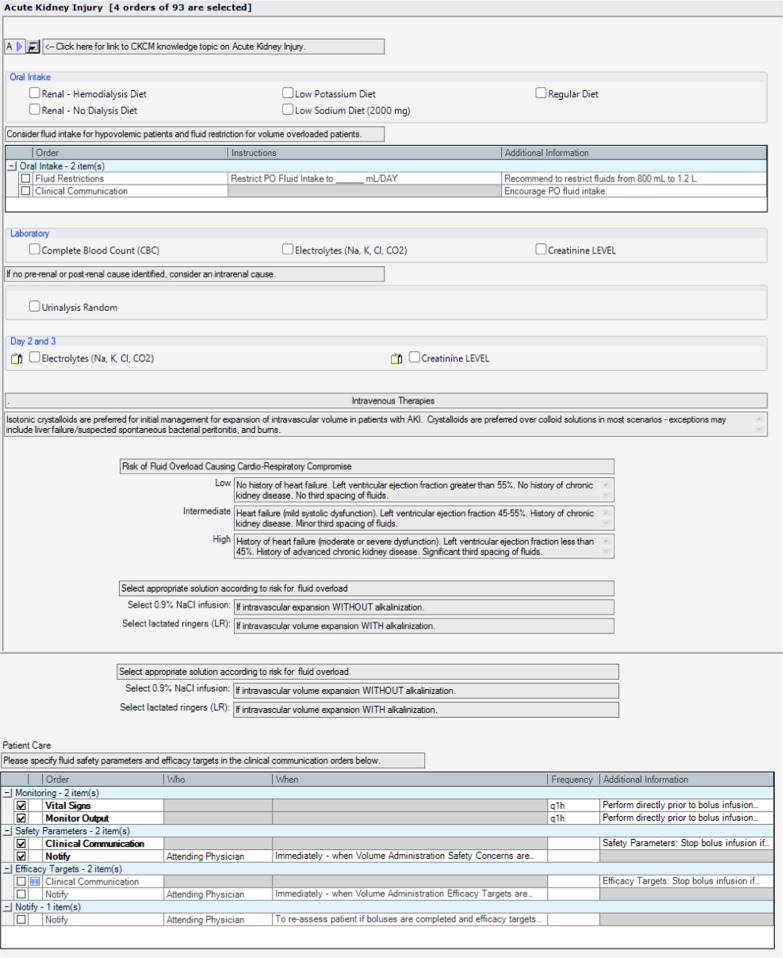

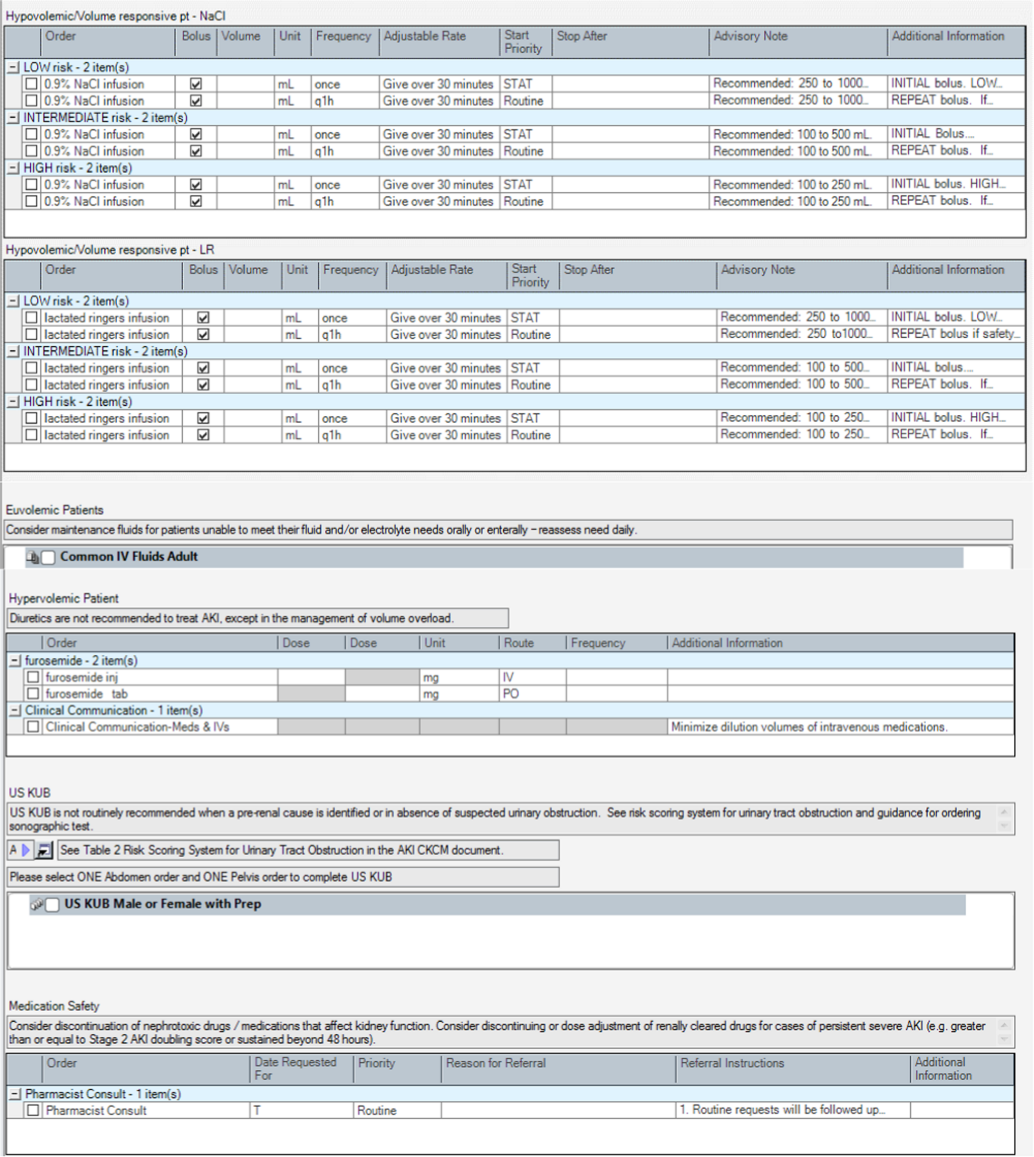

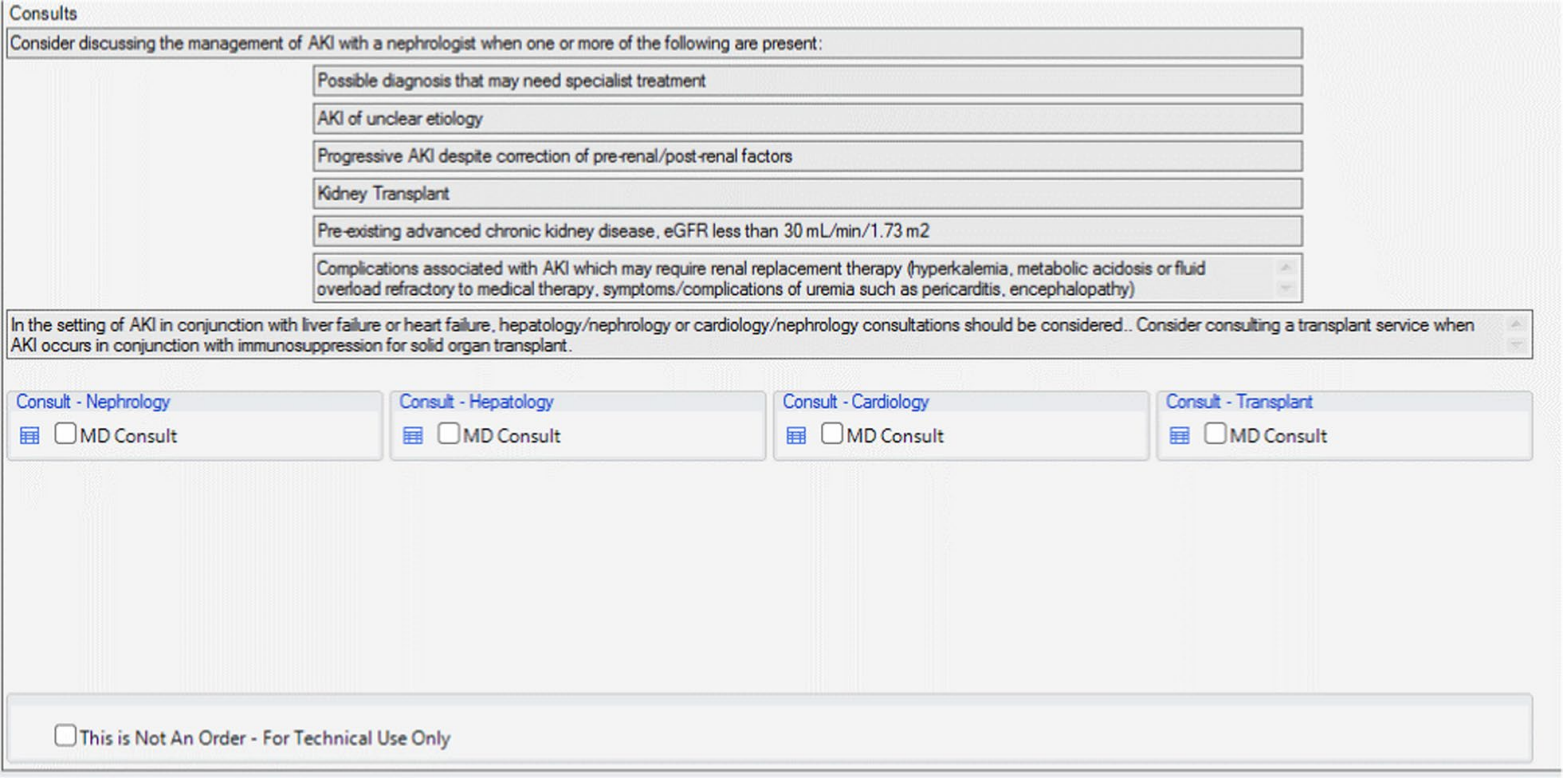
Acute kidney injury order set. AKI: acute kidney injury, USKUB: kidney, ureter, bladder ultrasound, IV: intravenous, ARB: angiotensin II receptor blockers, ACE: angiotensin converting enzyme, NSAIDs: non-steroidal anti-inflammatory drugs

The AKI order set provided a template for electronic order entry for investigation and management of patients with AKI according to guideline-based recommendations. The use of the AKI order set was designed to standardize investigations and tailor management to clinical assessment. The order set included orders for laboratory and diagnostic tests for patients with AKI, including a link to clinical guidance on when ultrasonography should be considered for investigation of urinary tract obstruction [36]. Orders for management were structured based on clinical assessment of patients as hypovolemic (potentially fluid/volume responsive), euvolemic, and hypervolemic (fluid/volume overloaded). This would link users to guidance on use of intravenous (IV) fluid boluses for patients identified as hypovolemic, use of maintenance IV fluids for patients identified as euvolemic, and use of diuretics for patients assessed as hypervolemic. The order set provided additional guidance for prescribing IV fluid boluses, including risk assessment for developing volume overload and appropriate specification of monitoring safety parameters to identify those developing volume overload with fluid resuscitation. Additionally, there were options to receive additional pharmacy support with management of medications for AKI patients and guidance on when consultation with nephrologists and other specialists for AKI was appropriate. The complete order set is shown in Fig. 5.

### Assessment of functionality, usability, and acceptability of the electronic clinical decision support tools

The Alberta Health Services Clinical Decision Support Configuration and Development Team conducted functional testing of the AKI stage alerts to ensure that the requirements were properly satisfied by the application in the SCM™ EMR (Additional file 1: Table S2). Following functional testing, the AKI alerts were implemented silently over a 30-day observation period for patients on 14 general medicine and surgery (general surgery, vascular surgery, and trauma surgery) units across four Calgary Zone hospitals. The alerts were activated in the background without being reported on the user display of the SCM™ EMR, to determine the frequency of the generated alerts and to characterize the content of the alerts including the total number of alerts generated and the number corresponding to each stage of AKI, the active medications included in the AKI alerts, and the patient location where AKI alerts were generated. Silent alerting for adverse medication warnings was assessed based on the total number of alerts generated, the AKI stage at the time of the medication warning and the medication order that prompted the AKI adverse medication warning.

Following the silent alerting phase, the AKI alerts and clinical summary dashboard were deployed as front facing applications to users of the SCM™ EMR on 14 Calgary Zone hospital units. Healthcare providers were oriented to the tools via an Alberta Health Services email notification and education sessions held for staff on each unit. Further, presentations on the AKI CDS initiative were held at the Calgary Medical Grand Rounds, Surgery Strategic Clinical Network meetings, General Surgeons’ business meetings, Vascular surgery rounds, Internal Medicine residency program academic half-day, and Surgery residency academic half-day. The usability of the AKI alerts, AKI adverse medication warnings, and clinical summary dashboard was evaluated using a 10-question survey measured using a 5-point Likert scale adapted from the System Usability Scale (SUS) developed by Brooke (Digital Equipment Corporation) [37]. Following initial implementation, the survey was distributed via email to nursing staff, pharmacists and physicians providing care on the units where the AKI CDS tools were available in order to evaluate acceptability and refine CDS tools as required. Participation in the survey was voluntary.

Formal user acceptance testing (UAT) of the AKI order set was conducted separately to assess the domains of efficiency, margin of error, learnability and satisfaction. User acceptance testing was done in collaboration with clinical user support experts from Alberta Health Services. The evaluation questionnaire is available in Additional file 1: Table S3.

The user acceptance testing consisted of an in-person questionnaire evaluating various aspects of usability and think-aloud testing to collect verbal feedback from physicians and nurses as they worked through various pre-designed clinical scenarios using the order set. Five different scenarios were designed with varying patients that allowed the users to experience use of the new order set in a testing environment on SCM™. Physicians were asked to enter orders based on the patient case scenarios and nursing staff were asked to evaluate how well they were able to understand the orders once the physicians had entered them through the order set.

## Results

### Functional testing of AKI stage alerts

Seventeen unique scenarios of alerting were tested by feeding the system input and verifying the output of the alerts (Additional file 1: Table S1). The functional testing showed that the alerts were appropriately generated in all cases when the AKI criteria were met and verified the EMR module was programmed correctly.

### Evaluating features of silent AKI alerts and AKI adverse medication warnings

There were a total of 81 AKI alerts generated; 67% were Stage 1 AKI, 17% were Stage 2 AKI, and 16% were Stage 3 AKI (Table 2). Of these AKI alerts, 44% had listed active medications that could reduce the patient’s kidney function, maintaining that this medication list feature will be frequently populated to inform care providers of relevant adverse medications that could be suspended or discontinued following AKI onset.

**Table 2:** Characteristics of acute kidney injury alerts generated in the SunRise Clinical Manager electronic medication record from the silent alert phase on 14 medical and surgical hospital units in the Calgary Zone over a 30 day observation period

Alert Frequency	Number (%)
Total AKI alerts	81 (100.0)
Stage 1 AKI alerts	54 (66.7)
Stage 2 AKI alerts	14 (17.3)
Stage 3 AKI alerts	13 (16.0)
Active medications included in AKI alerts	36 (44.4)
Diuretics	19 (23.4)
Antibiotics	1 (1.2)
ACE-I/ARB	10 (12.3)
NSAIDs	6 (7.4)
Patient location where AKI alert generated	
Medical unit	66 (81.5)
Surgical unit	15 (18.5)

*AKI* acute kidney injury, *ACE-I* angiotensin converting enzyme inhibitors, *ARB* angiotensin receptor blockers, *NSAID* non-steroidal anti-inflammatory drugs

There were 21 AKI adverse medication warnings during the silent phase (Table 3). Diuretics were the medication that most frequently generated the warning (52.4%), followed by antibiotics (19%), non-steroidal anti-inflammatory drugs (NSAIDs) (14%), and angiotensin converting enzyme inhibitors (ACEi) or angiotensin II receptor blockers (ARBs) (14%). Given the low frequency of this medication warning, it was maintained as an interruptive alert.

**Table 3:** Characteristics of acute kidney injury adverse medication warnings generated in the SunRise Clinical Manager electronic medication record from the silent alert phase on 14 medical and surgical hospital units in the Calgary Zone over a 30-day observation period

Alert Frequency	Number (%)
Total AKI adverse medication warnings	21 (100.0%)
AKI stage at time of adverse medication warning	
Stage 1 AKI alerts	15 (71.4)
Stage 2 AKI alerts	2 (9.6)
Stage 3 AKI alerts	4 (19.0)
Medication orders prompting AKI adverse medication warning	
Diuretics	11 (52.4)
Antibiotics	4 (19.0)
ACE-I/ARB	3 (14.23)
NSAID	3 (14.3)
Medical unit	10 (47.6)
Surgical unit	11 (52.4)

*AKI* acute kidney injury, *ACE-I* angiotensin converting enzyme inhibitors, *ARB* angiotensin receptor blockers*, NSAID* non-steroidal anti-inflammatory drugs

### Acceptability and refinement of AKI alerts, AKI adverse medication warnings, and AKI clinical summary dashboard

There were 23 respondents including 15 nursing staff, 3 pharmacists, and 5 physicians (Table 4). Of the respondents, 18 (78%) agreed that they would like to use the AKI decision support tools in SCM™ in their practice. There were 17 respondents who agreed the tools were easy to use and that they could be learned quite quickly. Eight participants (35%) indicated that they would want to learn more before they could use the tools appropriately, and 8 (35%) participants were undecided on whether they felt confident using the tools. Overall, the responses ranged from undecided to positive, and there were no negative perceptions towards the developed SCM tools. Undecided responses were largely from respondents who were unfamiliar with the tools and provided this information in a general comments section, suggesting that the tool design was accepted by care providers, but broader education was required to integrate the use of the electronic tools into practice. Complete results of this survey are reported in Table 5.

**Table 4:** Participant characteristics for usability survey evaluating clinical decision support tools

Age (n, %)	
< 30 years	6 (26%)
30–39 years	13 (56%)
40–49 years	2 (9%)
50–59 years	2 (9%)
Sex (n, %)	
Female	19 (83%)
Male	4 (17%)
Clinical role (n, %)	
Nursing staff	15 (65%)
Physician	5 (22%)
Pharmacist	3 (13%)
Number of years in practice (n, %)	
Less than 5 years	7 (30%)
5–10 years	9 (39%)
More than 10 years	7 (30%)

**Table 5:** Results of usability survey evaluating clinical decision support tools

	Strongly disagree N (%)	Disagree N (%)	Undecided N (%)	Agree N (%)	Strongly agree N (%)
I think that I would like to use the AKI care pathway and decision support tools in SCM in my care^a^	0 (0)	0 (0)	4 (18)	11 (50)	7 (32)
I found these tools in SCM unnecessarily complex	6 (26)	8 (35)	7 (30)	2 (9)	0 (0)
I found the tools were easy to use	0 (0)	1 (4)	4 (17)	11 (48)	6 (26)
I think that I would need assistance to be able to use the tools^a^	4 (18)	11 (50)	4 (18)	4 (18)	0 (0)
I found the various functions in these tools were well integrated	0 (0)	2 (9)	10 (43)	9 (39)	2 (9)
I thought the display was too confusing	3 (13)	12 (52)	8 (35)	0 (0)	0 (0)
I would imagine that most people would learn to use these tools very quickly	0 (0)	0 (0)	6 (26)	14 (61)	3 (13)
I found these tools very cumbersome/awkward to use	8 (35)	8 (35)	7 (30)	0 (0)	0 (0)
I felt very confident using these tools	0 (0)	1 (4)	8 (35)	10 (43)	4 (17)
I need to learn more before I could use the tools appropriately	5 (21)	6 (26)	4 (17)	8 (35)	0 (0)

*SCM* Sunrise Clinical Manager™

^a^Results reported for 22 participants—1 participant did not respond to the question

### User acceptance testing and modification of the AKI order set

**Fig. 6:**
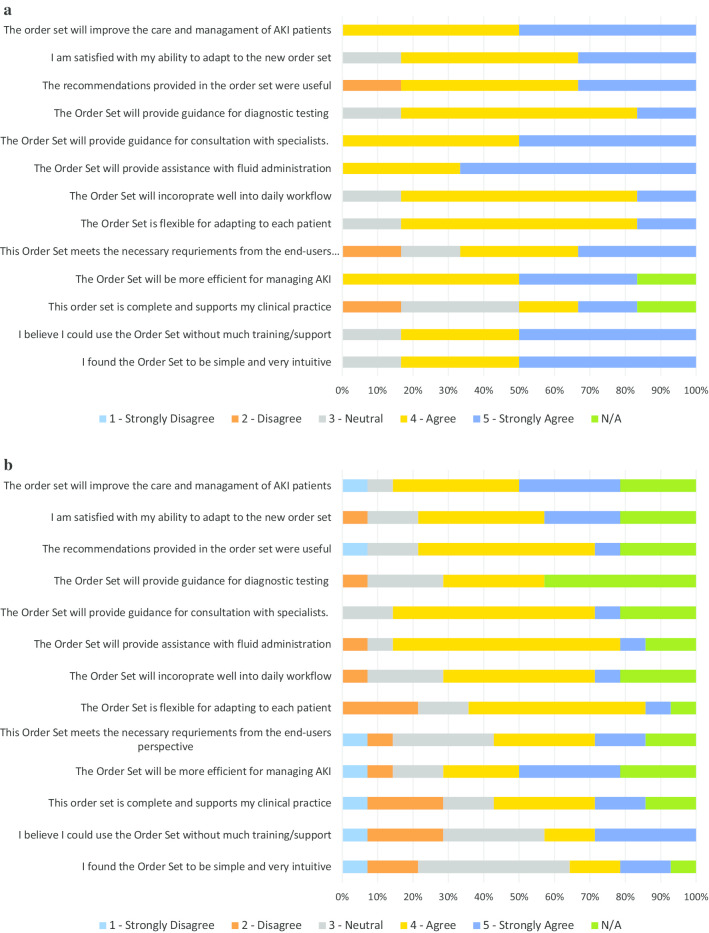
**a** Order set user acceptance testing results among physicians. **b** Order set user acceptance testing results among nursing staff

There were 6 physicians and 14 nursing staff who completed the UAT evaluation. The majority of the participants indicated that the order set helped with prescribing IV fluids (88%, 16/18), and with identifying appropriate indications for specialist consultations (88%, 15/17). Of the end-users that provided feedback, 76.5% (13/17) of the testers also agreed or strongly agreed that the recommendations were useful. Overall, 88% (15/17) believed that the order set would improve the care and management of AKI patients. There were some missing data on the questionnaires, as some nursing staff indicated that certain statements pertained mainly to the physicians and preferred not to respond. The quantitative results are presented in Fig. 6a and b. Comments during testing indicated that because the alerts and tools were built into the existing EMR, the alerts and following order set fit well into daily workflow and facilitated the ease of use on the units.

All of the physicians agreed that the order set would improve the care and management of AKI patients. Most physicians and nursing staff agreed that the order set would provide assistance with fluid administration. For physicians and nursing staff, the lowest rated category was the completeness of the order set. Nursing staff further indicated that the order set would be difficult to use without much training. Verbal feedback was sought on the lowest rated categories, which identified that there was a lack of clarity and information regarding monitoring safety and efficacy for patients receiving IV fluid boluses. There was also ambiguity in the order set language noted when nursing staff reviewed the submitted orders. Based on this feedback from testing participants, modifications to the order set were incorporated to ensure orders for IV fluid boluses and patient monitoring were clearly defined within the order set for physicians and could be clearly interpreted by nursing staff.

## Discussion

We developed a CDS initiative to improve clinical recognition and processes of care for AKI management, using a process of stakeholder engagement, development and functionality testing, and end-user evaluation of content, usability, and acceptability. The electronic tools developed include AKI stage alerts, AKI adverse medication warnings, AKI clinical summary dashboard, and order set. Involving end-users at all stages, including development of the content and design of the tools, allowed us to design tools that are expected to integrate more easily into clinical workflow and use in practice. Functional and usability testing helped resolve technical issues with alert functions and identified areas of improvement for the order set by seeking feedback directly from clinical end-users.

This CDS intervention was designed with the intention of avoiding alert fatigue while still maintaining that alerts could be made available and identified in a time-sensitive manner. Our intention was to create AKI alerts accompanied by a dashboard for monitoring AKI patients and an order set with guidance on management. Further, the goal was to develop these decision support tools within the existing SCM™ EMR system to improve integration into clinical practice. Previous work by Kawamoto et al*.* [14] identified primary features for successful CDS tools: the automatic provision of decision support as part of clinical workflow, the provision of decision support at time and location of decision making, and the provision of recommendation rather than just an assessment computer based generation of decision support. Our CDS tools for AKI were developed to adhere to all of these recommendations, with the aim to promote successful uptake by clinical end-users.

Haase et al. [8] conducted a systematic review on electronic alerts for AKI and found variability in effectiveness for clinical outcomes. Our intervention aligns with many aspects of successful interventions, such as the development of AKI alerts that are linked with warnings of potentially harmful medications, links to treatment recommendations, and the non-disruptive function of AKI alerts. Our tools included both non-disruptive and interruptive alerts with consideration of what information was pertinent at the point of care. Alert fatigue can contribute to unsuccessful implementation of alerts as the overwhelming number of notifications causes each subsequent alert to have less of an effect for the receiver of the alert. The use of KDIGO criteria [18] for creating the algorithm that triggers an initial AKI alert was a common criteria amongst other studies [11–13, 21, 32]. Multiple studies also included a link to bundled treatment recommendations [9–13], but had variable outcomes potentially due to varying uptake of the tools. While building our intervention, we considered which components have been shown to be effective in the literature while aiming to tailor the CDS tools within the existing EMR to increase accessibility.

The electronic CDS tools for AKI were designed for implementation on surgical and medical units of hospitals in Calgary Zone of Alberta Health Services, Alberta, Canada. Implementation requires individual tailoring of education and implementation strategies for each unit using approaches that work best for each specific unit due to the diversity of roles across different hospital units, and because the electronic tools require a user to interact with the system to prompt a response.

### Strengths and limitations

The AKI alerts, medication warnings, dashboard, and order set were integrated within the common hospital EMR, and healthcare providers were already familiar with the technical aspects of the system. As they were implemented within an existing EMR, all tools were also accessible at the point of care. However, the AKI alert flag appeared on the SCM front screen and required a member of the care team to select it to access information on AKI recognition. Consideration of alert fatigue helped aid the design of the intervention to be as acceptable as possible, so as to minimize disruption with other clinical tasks involving the EMR [38]. However, the requirement to review alerts manually could delay the response to AKI when first identified. Because the CDS tools require human interaction to initiate a response, the corresponding implementation plan requires the development of unit-specific protocols and designation of roles for various care providers including reviewing flag alerts. This will allow each unit to develop their own strategies for uptake of the new CDS tools. This is also expected to standardize AKI recognition through alerts and increase awareness among all care providers on the units.

Our AKI decision support intervention approach may be limited by requiring a behavioral adjustment to facilitate a change in the recognition and management of AKI [1]. In order to sustain the uptake of the SCM tools following implementation in hospitals where turnover of staff and resident physicians is frequent, continuous engagement will be required to maintain awareness on availability of tools to optimize their use. Education to nursing staff, physicians, and pharmacists when appropriate, will be important to facilitate successful uptake of the CDS tools into clinical care. This will be conducted through one-on-one and small group presentations on how to access and use the electronic CDS tools for AKI care.

The AKI alerts are generated based exclusively on serum creatinine, although the KDIGO criteria include urine output as well [18]. Urine output was intentionally not included in the generating of alerts as the recording of urine output can vary greatly between patients and hospital units and is prone to measurement error on hospital wards. For this reason, the alerts were limited to serum creatinine values that are more routinely ordered for hospital patients and can be more accurately compared to KDIGO criteria. This alerting function is consistent with many of the previously reported studies due to the challenges in urine output records for identifying AKI [8]. A novel aspect of our CDS system was the inclusion of guidance to tailor IV fluid therapies based on the assessment of a patient’s risk for volume overload and the incorporation of steps for monitoring the response to IV fluid administration, which may improve the effectiveness and safety of IV fluid administration in clinical care. Finally, a limitation of our study also includes the voluntary nature of our survey to assess the acceptability of the electronic CDS tools, which may be vulnerable to reporting bias.

## Conclusions

We designed a collection of CDS tools for AKI recognition and management including AKI stage alerts, adverse medication warnings, an AKI clinical summary dashboard, and an AKI order set. The tools were developed to improve early recognition and timely management of AKI in hospitalized patients on surgery and medical units. Evaluation and testing with clinical end-users resulted in minor modifications, and suggested that end-users found the tools acceptable and easy to use. Future work evaluating implementation of the AKI tools in the Calgary EMR will be required to determine whether they improve processes of care for AKI and patient outcomes. Implementation of the tools is expected to be an iterative process that can result in further modifications to the tools, based on ongoing feedback from health providers.

## Supplementary information

**Additional file 1.** Supplementary material.

## Data Availability

All data generated during this study are included in this published article and its supplementary information files.

## Abbreviations

AKI: Acute kidney injury
CDS: Clinical decision support
KDIGO: Kidney Disease Improving Global Outcomes
SCM™: Sunrise Clinical Manager™
UAT: User acceptance testing
