# Supplementary material for: Development and initial implementation of electronic clinical decision supports for recognition and management of hospital-acquired acute kidney injury
Source: BMC Med Inform Decis Mak. 2020 Nov 4;20:287. doi: 10.1186/s12911-020-01303-x (PMC7640650; doi:10.1186/s12911-020-01303-x)
Supplement: Supplementary file 1 — Additional file 1. Supplementary material. [file 12911_2020_1303_MOESM1_ESM.docx]

**Supplementary Material:**

**Table S1.** List of medications that could adversely affect kidney function following AKI

**Medications that affect kidney function or are nephrotoxic (consider discontinuing in all stages of AKI)**

| **NSAIDS** |
| --- |
| celecoxib cap |
| diclofenac / misoprostol 50 tab |
| diclofenac / misoprostol 75 tab |
| diclofenac EC tab |
| diclofenac SR tab |
| diclofenac sup |
| ibuprofen tab |
| ibuprofen liquid |
| indomethacin cap |
| indomethacin inj |
| indomethacin liquid |
| indomethacin supp |
| ketorolac inj |
| naproxen tab |
| naproxen liquid |
| naproxen supp |
| **DIURETICS** |
| acetaZOLamide tab |
| acetaZOLamide inj |
| acetaZOLamide liquid |
| chlorthalidone tab |
| aMILoride tab |
| aMILoride liquid |
| hydrochlorothiazide tab |
| hydrochlorothiazide / aMILoride tab |
| hydrochlorothiazide / triamterene tab |
| hydrochlorothiazide liquid |
| ethacrynic acid inj |
| ethacrynic acid liquid |
| ethacrynic acid tab |
| furosemide tab |
| furosemide infusion |
| furosemide inj |
| furosemide liquid |
| indapamide tab |
| metoLAZONE liquid |
| metoLAZONE tab |
| spironolactone tab |
| spironolactone / hydrochlorothiazide liq |
| spironolactone / hydrochlorothiazide tab |
| spironolactone liquid |
| mannitol |
| mannitol inj |
| **ACE INHIBITORS** |
| capTOPRIL tab |
| capTOPRIL liquid |
| cilazapril tab |
| enalapril tab |
| enalapril liquid |
| enalaprilat inj |
| fosinopril tab |
| lisinopril tab |
| perindopril tab |
| ramipril cap |
| trandolapril cap |
| **ANGIOTENSIN RECEPTOR BLOCKERS** |
| candesartan tab |
| irbesartan tab |
| losartan tab |
| telmisartan tab |
| valsartan tab |
| **CALCINEURIN INHIBITORS*** |
| cycloSPORINE cap |
| cycloSPORINE for dose adjustment |
| cycloSPORINE infusion |
| cycloSPORINE inj |
| cycloSPORINE liquid |
| tacrolimus cap |
| tacrolimus ER cap |
| tacrolimus infusion |
| tacrolimus liquid |
| *Consider therapeutic drug monitoring and adjusting the dose in consultation with transplant service for patients receiving calcineurin inhibitors in the setting of solid organ transplantation. |
| **ANTI-FUNGAL** |
| amphotericin B inj |
| amphotericin B lipid complex |
| amphotericin B LIPOSOMAL inj |
| amphotericin B syringe |
| **AMINOGLYCOSIDES** |
| amikacin inj |
| amikacin syringe |
| gentamicin inj |
| gentamicin liquid |
| tobramycin inj |

### **Medications requiring renal clearance that may require dose adjustment or discontinuation in greater than or equal to Stage 2 Acute Kidney Injury**

| **ANTIMICROBIALS** |
| --- |
| acyclovir tab |
| acyclovir inj |
| acyclovir liquid |
| amantadine tab |
| amikacin inj |
| amikacin syringe |
| amoxicillin / clavulanate tab |
| amoxicillin / clavulanate liquid |
| amoxicillin cap |
| amoxicillin CHEW tab |
| amoxicillin liquid |
| ampicillin inj |
| ceFAZolin 1 g / metroNIDAZOLE 500 mg inj |
| ceFAZolin 2 g / metroNIDAZOLE 500 mg inj |
| ceFAZolin 500mg/metroNIDAZOLE 250mg inj |
| ceFAZolin inj |
| cefePIME inj |
| cefixime tab |
| cefixime liquid |
| cefotaxime inj |
| cefOXitin inj |
| cefTAZidime inj |
| cefTAZidime syringe |
| cefUROXime tab |
| cefUROXime inj |
| cefUROXime liquid |
| cephaLEXIN tab |
| cephaLEXIN liquid |
| ciprofloxacin tab |
| ciprofloxacin inj |
| ciprofloxacin liquid |
| clarithromycin ER tab |
| clarithromycin tab |
| clarithromycin liquid |
| colistin inj |
| DAPTOmycin inj |
| ethambutol tab |
| ertapenem inj |
| erythromycin  EC cap |
| erythromycin estolate liquid |
| erythromycin inj |
| fluCONazole tab |
| fluCONazole inj |
| fluCONazole liquid |
| foscarnet inj |
| foscarnet syringe |
| ganciclovir inj |
| ganciclovir syringe |
| imipenem / cilastatin inj |
| levofloxacin tab |
| levofloxacin inj |
| levofloxacin liquid |
| meropenem inj |
| nitrofurantoin liquid |
| nitrofurantoin tab |
| oseltamivir tab |
| oseltamivir liquid |
| penicillin G benzathine inj |
| penicillin G sodium inj |
| penicillin V potassium tab |
| penicillin V potassium liquid |
| piperacillin / tazobactam inj |
| piperacillin inj |
| streptomycin inj |
| terbinafine tab |
| tetracycline cap |
| trimethoprim tab |
| sulfamethoxazole / trimethoprim   tab |
| sulfamethoxazole / trimethoprim  DS tab |
| sulfamethoxazole / trimethoprim  PED tab |
| sulfamethoxazole / trimethoprim inj. |
| sulfamethoxazole / trimethoprim liquid |
| trimethoprim liquid |
| valACYclovir tab |
| valACYclovir liquid |
| valGANciclovir  tab |
| valGANciclovir liquid |
| vancomycin infusion |
| vancomycin inj |
| vancomycin syringe |
| voriconazole inj |
| voriconazole syringe |
| ticarcillin / clavulanate inj |
| **CARDIAC** |
| digoxin tab |
| digoxin inj |
| digoxin liquid |
| nitroprusside infusion |
| quiNIDine bisulfate CR tab |
| **ANTICOAGULANTS** |
| apixaban tab |
| dabigatran cap |
| dalteparin inj |
| enoxaparin inj |
| eptifibatide infusion |
| eptifibatide inj |
| fondaparinux inj |
| rivaroxaban tab |
| tinzaparin inj |
| For patients receiving therapeutic LMWH who have a calculated creatinine clearance less than 30 mL/min), we suggest a reduction of the dose rather than using standard doses. |
| **HYPOGLYCEMICS** |
| acarbose tab |
| glyBURIDE tab |
| GLICLAzide MR tab |
| GLICLAzide tab |
| metFORMIN tab |
| pioglitazone  tab |
| SITagliptin  tab |
| SAXagliptin tab |
| **OTHER** |
| allopurinol tab |
| allopurinol liquid |
| codeine tab |
| codeine CR tab |
| codeine inj |
| codeine liquid |
| colchicine tab |
| gabapentin tab |
| lithium carbonate cap |
| lithium citrate liquid |
| meperidine inj |
| meperidine PCA inj 10 mg/mL |
| methotrexate |
| metoclopramide |
| metoclopramide tab |
| metoclopramide infusion |
| metoclopramide inj |
| metoclopramide liquid |
| morphine tab |
| morphine  ER cap |
| morphine infusion |
| morphine inj |
| morphine liquid |
| morphine PCA inj |
| morphine PCA inj 5 mg/mL |
| morphine supp |

**Table S2.** Functional Testing for AKI alerts scenarios

AKI: acute kidney injury, C1: serum creatinine value that generated the AKI alert, MLM: Medical Logical Module RV: reference value of serum creatinine to assess change according to international Kidney Disease Improving Global Outcomes (KDIGO) criteria, ULRI: upper limit of reference interval

| **Test Case Name** | **Test Case Description** | **Expected Outcome** |
| --- | --- | --- |
| Stage 1 AKI Alert RV ratio >1.5 RV>53 | verify MLM that generates AKI 1 Alert if RV ratio > 1.5 and RV > 53 | AKI 1 Alert is displayed. |
| Stage 1 AKI Alert RV ratio >1.5 RV<53 | verify MLM that dose not generate any alert if RV is less than or equal to 53 | no AKI Alert displays |
| patient not in FMC102 or FMC26 | verify MLM that dose not generate any alert if patient is not on FMC102 or FMC26 | no AKI Alert displays |
| Stage 1 AKI Alert RV ratio >1.5 RV>53, patient has an active encounter in Hemo | verify MLM that dose not generate any alert if patient has an active encounter in Hemo units | no AKI Alert displays |
| patient has previous AKI 1 Alert before | verify MLM that dose not generate any alert if patient has previous AKI 1 alert | no AKI Alert displays |
| patient has AKI 1, then normal lab result, then AKI result again | verify MLM that displays both AKI alerts | after step 2: AKI 1 alert displays after step 3: no alert displays after step 4: AKI 1 alert displays |
| Stage 1 AKI Alert RV ratio<1.5  D>26 within 48 hours RV<100 for woman | verify MLM that generates AKI 1 alert if certain criteria meets requirement | after step 2: AKI 1 Alert is displayed.  after step 3: no alert displays |
| Stage 1 AKI Alert RV ratio<1.5  D>26 within 48 hours RV<120 for men | verify MLM that generates AKI 1 alert if certain criteria meets requirement | after step 2: AKI 1 Alert is displayed.  after step 3: no alert displays |
| Stage 1 AKI Alert RV ratio<1.5  D<26 within 48 hours | verify MLM that does not generate AKI 1 alert if certain criteria do not meet the requirement | No Alert displayed. |
| Stage 2 AKI Alert RV ratio >2.0 RV>53 patient has active nephrotoxin on chart | verify MLM that generates AKI 2 Alert , as well as active nephrotoxin on chart | AKI 2 Alert is displayed with the name of the celecoxib item showing in the alert wording. |
| Stage 3 AKI Alert RV ratio > 3 | verify MLM that generates AKI 3 alert if certain criteria meets requirement | AKI 3 Alert is displayed. |
| Stage 3 AKI Alert there is previous AKI3 alert | verify MLM that does not generate AKI 3 alert if there is previous AKI 3 alert | No Alert displayed. |
| Stage 3 AKI Alert there is previous AKI2 alert | verify MLM that generates AKI 3 alert if there is previous AKI 2 alert | AKI 3 Alert is displayed. |
| Stage 3 AKI Alert adult patient C1>354 | verify MLM that generates AKI 3 alert if certain criteria meets requirement | AKI 3 Alert is displayed. |
| Stage 3 AKI Alert ped patient C1>3ULRI | verify MLM that generates AKI 3 alert if certain criteria meets requirement | AKI 3 Alert is displayed. |

**Table S3.** User Acceptance Testing Evaluation Form

| **Rating Scale Definition:** | | | | |
| --- | --- | --- | --- | --- |
| **1** | **2** | **3** | **4** | **5** |
| Strongly Disagree | Disagree | Neutral | Agree | Strongly Agree |

**Questionnaire:**

| 1 | I found the Order Set to be simple and very intuitive. | 1 | 2 | 3 | 4 | 5 |
| --- | --- | --- | --- | --- | --- | --- |
| 2 | I believe that I could use the Order Set without much training / support. | 1 | 2 | 3 | 4 | 5 |
| 3 | This order set is complete and supports my clinical practice. | 1 | 2 | 3 | 4 | 5 |
| 4 | The order set will be more efficient for managing AKI. | 1 | 2 | 3 | 4 | 5 |
| 5 | This Order Set meets the necessary requirements from the end-users perspective | 1 | 2 | 3 | 4 | 5 |
| 6 | The order set is flexible for adapting to each patient. | 1 | 2 | 3 | 4 | 5 |
| 7 | The order set will incorporate well into daily workflow. | 1 | 2 | 3 | 4 | 5 |
| 8 | The order set will provide assistance with fluid administration. | 1 | 2 | 3 | 4 | 5 |
| 9 | The order set will provide guidance for consultation with specialists. | 1 | 2 | 3 | 4 | 5 |
| 10 | The order set will provide guidance for diagnostic testing. | 1 | 2 | 3 | 4 | 5 |
| 11 | The recommendations provided in the order set were useful. | 1 | 2 | 3 | 4 | 5 |
| 12 | I am satisfied with my ability to adapt to the new order set. | 1 | 2 | 3 | 4 | 5 |
| 13 | The order set will improve the care and management of AKI patients. | 1 | 2 | 3 | 4 | 5 |

Comments:

****Think aloud testing was also conducted and verbal feedback was summarized into notes by research associates.***
